# Use of Natural-Fiber Bio-Composites in Construction *versus* Traditional Solutions: Operational and Embodied Energy Assessment

**DOI:** 10.3390/ma9060465

**Published:** 2016-06-13

**Authors:** Carmen Galan-Marin, Carlos Rivera-Gomez, Antonio Garcia-Martinez

**Affiliations:** Institute of Architecture and Construction Science, University of Seville, Seville 41012, Spain; crivera@us.es (C.R.-G.); agarcia6@us.es (A.G.-M.)

**Keywords:** life cycle assessment, operational energy, embodied energy, natural composites, natural fibers, sustainability, load bearing walls

## Abstract

During the last decades natural polymers have become more and more frequent to replace traditional inorganic stabilizers in building materials. The purpose of this research is to establish a comparison between the most conventional building material solutions for load-bearing walls and a type of biomaterial. This comparison will focus on load-bearing walls as used in a widespread type of twentieth century dwelling construction in Europe and still used in developing countries nowadays. To carry out this analysis, the structural and thermal insulation characteristics of different construction solutions are balanced. The tool used for this evaluation is the life cycle assessment throughout the whole lifespan of these buildings. This research aims to examine the environmental performance of each material assessed: fired clay brick masonry walls (BW), concrete block masonry walls (CW), and stabilized soil block masonry walls (SW) stabilized with natural fibers and alginates. These conventional and new materials are evaluated from the point of view of both operational and embodied energy.

## 1. Introduction

Spain is, among other countries, a signatory of the United Nations Commission on Sustainable Development (CSD). As stated by the organizing committee of the 2015 United Nations Climate Change Conference, COP 21 or CMP 11 that was held in Paris, from the outset of the talks, the expected key result was an agreement to set a goal of limiting global warming to less than two degrees Celsius (°C) compared to pre-industrial levels [1]. The construction sector has a significant impact on the environment [2]. According to some studies, around 20% of the total impacts are related to manufacturing, construction, demolition processes, and final disposal of building materials, elements and systems [3], but the operational energy is, in many cases, considered the most significant aspect.

Energy in buildings can be categorized into two types: firstly by energy for the maintenance/servicing of a building during its useful life, namely operational energy (O.E.) and, secondly, by energy capital that goes into production of a building using various building materials, named embodied energy (E.E.). Study of both types of energy consumption is required for a complete understanding of building energy needs. Embodied energy of buildings can vary over wide limits depending upon the choice of building materials and building techniques. Reinforced concrete walls, fired clay brick masonry, concrete block masonry, bream and block slabs represent common conventional systems forming the main structure of buildings in Spain. Similar building systems can be found in many other developed and developing countries. Alternative building technologies, such as stabilized soil blocks, can be used for minimizing the embodied energy of buildings [4,5,6,7,8,9,10]. Generally, the materials used for the structure of buildings represent more than 50% of the embodied energy in the building [11]. In this sense, the use of alternative materials, such as mortar/concrete blocks, stabilized soil blocks, or fly-ashes, instead of materials with a high embodied energy, such as reinforced concrete, could save 20% of the cumulative energy over a 50-year life cycle [12]. In addition, recycling building materials [13,14] is essential to reduce the embodied energy in the building. For instance, the use of recycled steel and aluminum saves more than 50% in embodied energy [15].

There are many studies in the literature dealing with energy analysis in buildings [16], some of them have been published thus far on the evaluation of environmental impacts using a life cycle assessment (LCA) tool on the operational energy in the life cycle of residential dwellings. These studies have appeared during the last 10 years [17,18]. Furthermore, in different countries some other LCA case studies for operational energy of residential dwellings were performed, as in the following examples:

Sartori and Hestnes [19] performed a literature survey on buildings’ life cycle energy use of 60 cases from nine countries. This study concluded that operating energy represents by far the largest part of energy demand in a building during its life cycle.

Ortiz *et al.* [20] have applied LCA methodology to evaluate environmental impacts on a Spanish Mediterranean house located in Barcelona with a total area of 160 m^2^ and a projected 50-year life span. The same authors studied [21] the importance of operational energy in life cycle assessment (LCA). The comparison was applied within the residential building sector for two buildings, one in a developed country (Spain), and one in a developing country (Colombia).

Chung *et al.* [22] conducted an energy input–output (E-IO) analysis in Korea. The results showed that accounting for energy intensities and greenhouse gas (GHG) emission intensities is becoming an essential step in proper understanding of the energy usage structure.

Thormark [23], in Sweden, analyzed, within its CEPHEUS project (cost efficient passive houses as European standard) in the European Thermie program, on how far the design phase of housing was relevant with regard to reducing operational energy and how the choice of building materials may affect both embodied energy and recycling potential during the 50-year life span of the building.

Stephan and Stephan [24] quantized the life cycle energy and cost requirements associated with 22 different energy-reduction measures targeting embodied, operational, and user-transport requirements. It evaluates a case study apartment building in Sehaileh, Lebanon.

Koesling *et al.* [25] established a model valid for various types of building sector and applied it to estimate the amount of embodied energy in the building envelopes of 20 dairy farms in Norway.

Guan *et al.* [26] studied the energy used in all three phases of construction, operation, and demolition of eight residential buildings in and around Brisbane, Queensland, Australia. It was found that the main contribution to the operational energy in residential buildings comes from the use of general appliances in homes.

Brown *et al.* [27] analyzed how to mitigate climate change through operational energy reduction in existing Swedish residential buildings.

Zhu *et al.* [28] developed a new optimization method for building envelope design in order to get the lowest carbon emissions of building operational energy consumption using an orthogonal experimental design.

Pinky and Sivakumar [29] presented a case study of life cycle energy analysis of a residential development consisting of 96 identical apartment-type homes located in Southern India. They considered that the life cycle energy of the building includes the construction energy, operational energy and demolition energy. Construction refers to initial construction as well as recurring maintenance and repair work.

Praseeda *et al.* [30] discussed the embodied energy and operational energy assessment of a few residential buildings in different climatic locations in India. The study shows that the balance of O.E. and E.E. in LCA greatly depends upon the types of materials used in construction and extent of space conditioning adopted. In some cases E.E. can exceed O.E. in the whole life cycle. Buildings with reinforced concrete frames and monolithic reinforced concrete walls have very high E.E.

Stephan *et al.* [31] presented a framework which takes into account energy requirements at the building scale, *i.e.*, the embodied and operational energy of the building and its refurbishment, and at the city scale, *i.e.*, the embodied energy of nearby infrastructures and the transport energy (direct and indirect) of its users. This framework has been implemented through the development of a software tool which allows the rapid analysis of the life cycle energy demand of buildings at different scales.

Islam *et al.* [32] described the life cycle assessment and life cycle cost analysis of a typical Australian house under the design phase in their paper. The implications of life cycle environmental impacts and life cycle costs were evaluated and the optimum assemblage design is reported using an optimization algorithm. A set of best solutions is found depending on different factors: the model assumptions, range of environmental and economic indicators considered, and the chosen quantitative criteria.

Iddon and Firth [33] developed a building information model (BIM) tool to simultaneously estimate embodied and operational carbon over a 60 year life span for a typical four bedroom detached house. Using the tool, four different construction scenarios are evaluated, representing a range of current construction methods used in present day house buildings in the UK.

Finally, Ibn-Mohammed *et al.* [34] took a retrospective approach to critically review the relationship between embodied and operational emissions over the lifecycle of buildings in their paper. This is done to highlight and demonstrate the increasing proportion of embodied emissions, which is a consequence of the efforts to decrease operational emissions.

The current study takes an environmental perspective when comparing various conventional technologies for building walls to others that use new low-impact materials. By identifying and quantifying the materials used in the manufacturing and construction processes and the consequent operational energy, by applying LCA methodology, we identify the environmental impact of each alternative building material studied. Therefore, the aim of this research is to compare the environmental aspects and potential impact associated with the construction, maintenance, use, and disposal of walls in three-storey buildings, determining the option with the lowest negative impact in relation to insulation and material characteristics. A life cycle assessment was made of three models of housing blocks erected with load-bearing walls that varied according to their material structure. The options compared involved conventional and unconventional building materials. 

There are several previous studies above mentioned comparing different structural system in terms of LCA, but tend to focus just in one of the aspects. This study implements three different kinds of parameters in a single case study, the structural comparison, the material comparison, and the environmental comparison. The last variable included is to compare the results in two real climate conditions and real scenarios.

The aim of this research is to determinate which material type produces the least environmental impact during the different stages of its life cycle. The purpose in this article is to demonstrate the relationship between materiality, architecture, and design, and the environmental impact produced by different construction systems. In order to establish this relationship, life cycle assessment is the tool used to analyze the environmental impact produced by the construction materials studied in its life cycle. 

The main limitations of the study are related to the variables used. Only two alternatives to the stabilized earth block have been selected, being the most widely used materials in this kind of load-bearing wall. A specific building type has been chosen as a case study, whereas this decision comes from the abundance of its use in Europe in the twentieth century. Two climate scenarios have been selected, the most different ones among the Spanish climate zones. Just one type of insulating material has been introduced, being the most used in this type of construction solutions. Previous studies, comparing three different insulation materials, provided no significant differences in results, increasing the complexity of the data presentation and understanding.

## 2. Case Study

### 2.1. Building Description

**Figure 1:**
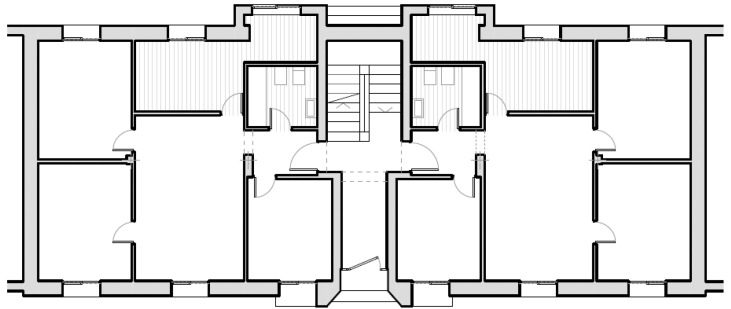
Building plan of the analyzed case study.

The case study is a typical apartment block, very common in the European scenario and a scheme widely used for social housing in the last century. The building is a three-storey case (maximum height allowed in social housing in Spain that does not require the introduction of an elevator) and a typical distribution of two apartments per floor (see Figure 1), balancing the use of the common space *versus* housing spaces. Surface area per floor is 147.83 m^2^, so the total built area of the building is 443.49 m^2^, with a total of six houses.

It is a mixed-structure building: façade load-bearing walls and inner concrete pillars. The floor slabs are a beam and block system and reinforced concrete beams, thus optimizing the structural function of the materials used in the envelope walls, while the pillars inside allow a free design of the interior plant.

**Figure 2:**
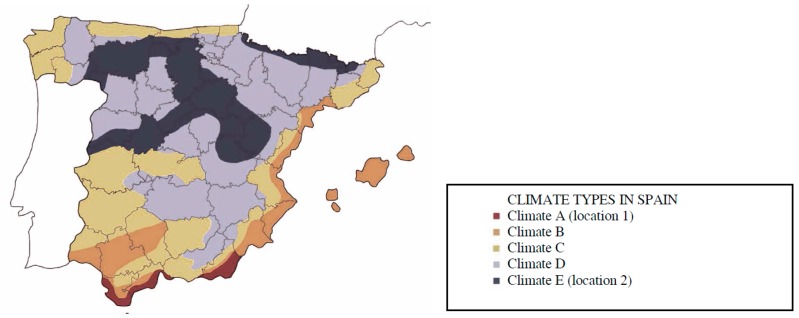
Climate types in Spain according to CTE-DB-HE [38].

In order to analyze the influence of the construction materials, several material options for the façade load-bearing walls have been studied. The different building construction systems used are BW (fired brick walls); CW (concrete block walls); and SW (stabilized soil walls). These systems are detailed in the next section. Inner concrete pillars are considered for all three cases. All of the ACV calculations are done according to ISO 14040 [35] and ISO 14044 [36], which implies a 50 year lifespan of the building. For the purpose of our study, the building is located in Spain. In order to analyze the influence of the operational energy demand, the building is located in two different Spanish climates (see Figure 2). These climates are named Location 1, corresponding to the Mediterranean climate and Location 2, corresponding to the inner continental areas of the peninsula.

The thicknesses of the load bearing walls have been calculated in order to mechanically respond to the loads of the different levels. The insulation thickness has been calculated for all three types of materials and both climates considered in order to meet the standards for the building envelope U values. The software used to establish the structure of the models proposed is CYPECAD. Madrid (Spain) (2015) [37], which is used for the analysis and design of building structures in homes, buildings, and civil engineering projects that have horizontal and vertical loads. This program is adapted to international regulations and automatically generates hypotheses for any user-defined combination according to the stated premises. Users can also define their own project situations to personalize the combinations to be taken into account in the calculations for the structural elements of the project. Entering data, such as the physical parameters of the different materials and the building characteristics, in the software gives us the wall dimensions. The results are displayed in Table 1.

According to Spanish regulations, CTE_DB-HE [38] the limitation for the building envelope U values are, for the warm Mediterranean climate (Location 1) zone A3: 0.94 W/m^2^·K, and for the continental climate (Location 2) zone E1: 0.57 W/m^2^·K. Polyurethane has been used as an insulation material and the thickness required varies between 2 and 4 cm.

### 2.2. Materials Used

To establish a standard of comparison, we have chosen common, and not so common, building materials widely used for a specific building typology. Such conventional materials are: fired clay brick masonry walls (BW) and concrete block masonry walls (CW). The unconventional material is a natural fiber bio-composite stabilized soil block, also used for masonry walls (SW). 

#### 2.2.1. Fired Clay Brick Walls (BW)

Fired bricks are among the oldest and most enduring of mankind’s building materials. The current fired bricks require a considerable amount of thermal energy during the burning process because they are fired at temperatures between 1000 °C and 1200 °C, depending on the clay. Light-colored clays usually require higher firing temperatures than dark-colored ones. This translates into a thermal energy of 3.75–4.75 MJ per brick. An average value of 4.25 MJ per brick (standard size in Spain: 240 × 115 × 70 mm^3^) has been considered for the comparison and computation of energy content of buildings and masonry.

#### 2.2.2. Concrete Block Walls (CW)

Concrete blocks are light-weight/low-density blocks very commonly used for the construction of envelope walls in multi-storey buildings in many countries. The basic composition of the blocks consists of cement, sand, and coarse aggregates (less than 4 mm in size). The energy content of the block will mainly depend on the cement percentage. Energy spent for crushing of coarse aggregate will also contribute to the block energy. The cement percentage generally varies between 7% and 10% by weight. The quality of the block, particularly its compressive strength, is the deciding factor for cement percentage. Energy content of the concrete block of size 400 × 200 × 200 mm^3^ will be in the range of 12.3–15.0 MJ.

#### 2.2.3. Stabilized Soil Block Walls (SW)

The stabilized soil blocks considered in this research are made by the combination of clay soil, water, a natural polymer as a stabilizer, and animal fiber reinforcement. The polymer used is calcium alginate, which is added to the mixture in the proportion of 1.2% by weight [39]. Calcium alginate production is a chemical synthesized from wet, chopped seaweed adding calcium chloride and sodium carbonate. The animal fiber is wool, used cut and raw, without washing or processing; the proportion used is 0.25% by weight. Blocks are cured at room temperature. The energy consumption is mainly by transport and extracting since they are not fired or steam cured. Barber and Pellow [40] reported results for the impact category of Energy Use for the on-farm phase of production for merino wool of 13.42 MJ per kg greasy wool or 22.55 MJ per kg wool fiber. The wool process gives the greatest uncertainty in energy use scouring and since this contributed almost 90% of the total processing. Processing was the single largest use of energy in the supply chain but the authors recognize the great variability in the results for the processing phase. For the block manufacturing, the wool used is unprocessed raw material (see Table 2).

## 3. Life-Cycle Assessment Goal and Scope

The particular focus of the application of the life-cycle assessment (LCA) in this study is to obtain the values of the embodied energy and global warming potential impacts (GWP) categories associated with the construction of three types of bearing walls: fired clay brick masonry walls (BW), concrete block masonry walls (CW), and stabilized soil block masonry walls (SW). A three storey construction is evaluated. To establish a suitable comparison framework, after calculating the necessary thickness of the walls, thermal conductivities have been unified in order to obtain equivalent operational energy parameters for all three materials.

According to the proposed framework, this study should answer the following question: what are the impacts produced by the processes related to the construction for each one of the combinations proposed?

According to the objective of this study the functional unit established is the total surface of walls in each case.

The assessed system is composed of every process that takes part in the production, construction, maintenance, deconstruction, and final disposal of every component of the building structure. It has excluded every process related to the operational phase of the dwelling. The system includes the following processes:

- Manufacturing of the building products phase. For each building material involved in the building every good and service from cradle to grave are considered. The manufacturing of employed machinery and territorial infrastructure processes has been considered.
- Assembly and construction phase. This covers every process aimed at integrating all products and services in the site in each studied dwelling. The transportation of building materials from the factory to the site, the placement of building products has been considered
- Maintenance and repair phase. This includes all repair operations and maintenance of building components. The renewal of those materials which have a lower durability has been considered.
- Dismantling and demolition phase. Every process carried out at the end of the life of the building to remove and demolish the dwelling has been taken into consideration: demolition, removal of building elements, and transportation of demolition materials to recycling or disposal have been included.
- Disposal and recycling phase. This covers all processes suffered by demolition materials after dismantling *i.e.*, the deconstruction of building materials.

The environmental data of wool and algae have been extracted from the recent studies conducted by Barber and Pellow [40] and Resurreccion *et al.* [41], respectively. The environmental data of the rest of the building materials were obtained from the well-recognized ECOINVENT database [42]. The calculation procedure to obtain the life cycle inventory was the described by García-Martínez [43]:

(1) Identification and quantification of the initial building products and auxiliary materials—including replacement materials that take part in the life cycle.
(2) Identification and quantification of the basic processes associated with the construction and deconstruction. The determination of the energy consumed during the construction and demolition is obtained as a factor of the total building material volume, following the procedure as described by Kellenberger *et al.* [44]. The following procedure has been taken:
  (a) Basic materials have been grouped into unit processes (Table 3). Construction systems, structural elements, walls and roofs, windows, doors, and finishing materials (from floors, ceiling, and walls) has been considered. The dimensioning of these elements is according to the obtained structural and thermal values (Table 1).
  (b) Division into groups and listing product specifications. Each case study has been divided into building elements according to the Building Cost Data Base of Andalusia (BCCA). The materials, the building machinery, and the labor has been related to each building element.
  (c) The building elements have been quantified using the construction management software Presto V.8 by RIB Software AG, Stuttgart (Germany).
  (d) The basic materials used in each case study has been obtained from the results given by Presto.
  (e) Once quantify each basic material and considering its physics properties, its mass and volume has been obtained.
(3) Determination of input and output of each unit process. The ECOINVENT database and published LCA studies have been used to obtain environmental information of unit processes (see Table 3). Final disposal processes for the plastics, metals, bitumen-based and wood materials have been considered. Other materials have been considered inert from the point of view of their final disposal. The quantification of the final disposal processes has been obtained from the quantities the initial basic products (Table 4).
(4) Inventory and assessment. The impact assessment is carried out using the CML 2001 method in relation to the GWP impact category. The “cumulative energy demand” in relation to the embodied primary energy (Table 3).
(5) Operational energy data are considered according to the benchmarks included in the Spanish ministry report SPAHOUSE [45] for houses located in Spain, considering two of the climates mentioned, location 1, corresponding to the warm Mediterranean climate and location 2, corresponding to the inner continental areas of the peninsula. Including insulation, all building envelope U values are considered equal for all three materials and subsequently, the operational energy will be considered to be the same for all three cases. Insulation is placed inside the building envelope so as to minimize the differences of thermal inertia among them.

## 4. Results

The results of the inventory list of the construction materials used for every type of construction system and climate are shown in Table 4.

The following phases have been considered for the LCA: manufacturing, (PH1); construction/demolition (PH2); transport (PH3); final disposal (PH4); operational energy (PH5). Table 4 shows the inventory list of the construction materials used for every stage, type of building construction system, and climate.

**Figure 3:**
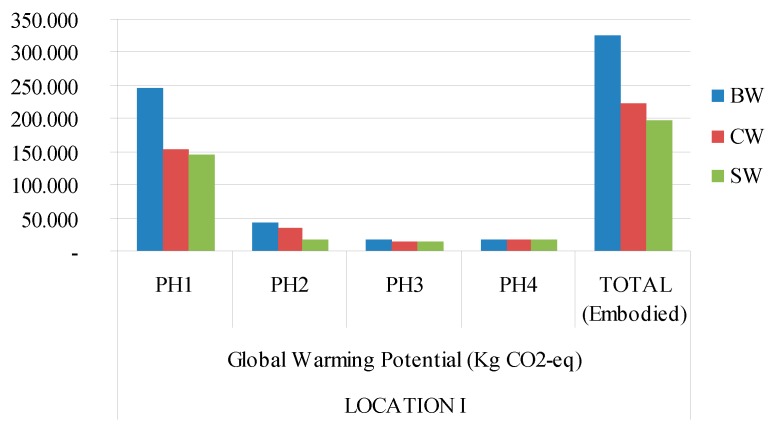
Global Warming Potential (GWP) 100a (Kg eq-CO_2_) for each type of building construction system and Mediterranean climate (location 1). PH1: manufacturing; PH2: constructing−demolishing; PH3: transport; PH4: final disposal. BW: ceramic brick walls; CB: concrete block walls; SW: stabilized soil walls.

**Figure 4:**
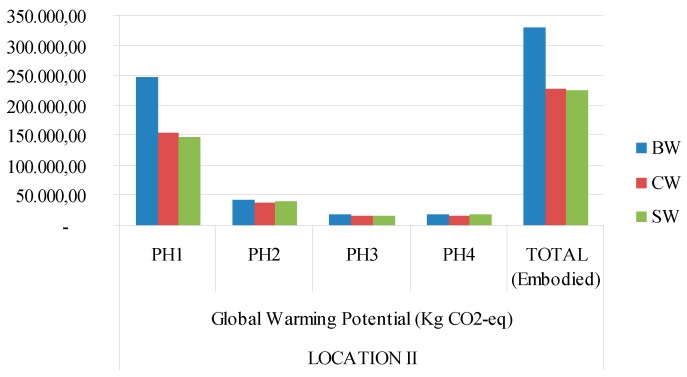
GWP 100a (Kg eq-CO_2_) for each type of building construction system and continental climate (location 2). PH1: manufacturing; PH2: constructing−demolishing; PH3: transport; PH4: final disposal. BW: ceramic brick walls; CB: concrete block walls; SW: stabilized soil walls.

**Figure 5:**
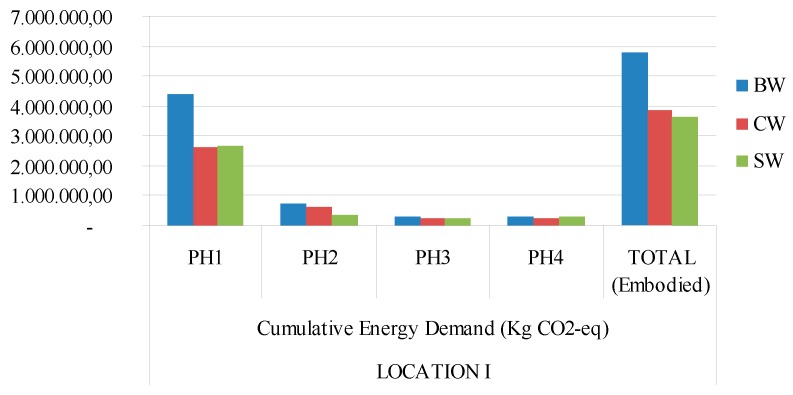
Cumulative energy demand (MJ) GWP 100a (Kg eq-CO_2_) for each type of building construction system and continental climate (location 1). PH1: manufacturing; PH2: constructing−demolishing; PH3: transport; PH4: final disposal. BW: ceramic brick walls; CB: concrete block walls; SW: stabilized soil walls.

**Figure 6:**
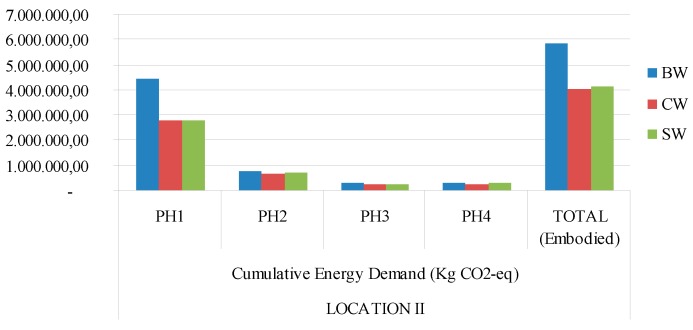
Cumulative energy demand (MJ) GWP 100a (Kg eq-CO_2_) for each type of building construction system and continental climate (location 2). PH1: manufacturing; PH2: constructing−demolishing; PH3: transport; PH4: final disposal. BW: ceramic brick walls; CB: concrete blocks walls; SW: stabilized soil walls.

Figure 3, Figure 4, Figure 5 and Figure 6 show a graphic comparison for embodied energy values and the global warming potential of all three types of materials, with both locations considered.

Table 5 shows an extensive list of environmental impact values shown by type of building construction system and climate location that allow a comparison of embodied *versus* operational energy in different materials and locations. Results are given globally for the entire building, per square meter and year.

## 5. Discussion

Regarding cumulative energy demand and global warming potential (GWP), for the different LCA phases, a proportion can be seen between the consumption of the three different materials employed. Manufacturing phase PH1 is the most relevant phase for embodied energy for both CO_2_-eq and MJ. The contribution of the manufacturing phase to these results is significant, representing mean percentages in relation to the total stages. In this phase there are significant differences that make the brick wall a more unfavorable material from energy consumption and emissions associated point of view, being more than 65% higher than the stabilized soil wall. The values of both soil and concrete block walls are quite similar. Nevertheless, in all cases the embodied energy is lower for the SW than for the other two materials except for the cumulative energy demand of location 2 due to greater energy employed in soil transportation.

Taking into account the different locations, there are no big differences between the values of embodied energy for both climates. The differences between materials are higher in global warming potential than in the cumulative energy demand. 

If different phases are compared, both CO_2_ emissions and cumulative energy demand, the greatest differences among both climates takes place in PH2. Analyzing Table 5, consumption data per m^2^ and year, they show remarkable differences between SW and BW values of total embodied energy. CO_2_ emissions for BW rise up to 1.6 times higher in cold climates. For warm climates, BW exceeds 1.5 times the values of SW.

The operational energy emissions of the building are higher than the ones associated with the embodied energy for all three materials. Accordingly the operational energy emissions of the building represent more than 200% of the embodied energy for the SW case. However for BW operational energy represents only 130% of embodied energy. 

Accordingly with these results it could be said that the energy consumed to build houses with brick walls in warm climates represents 165% of the energy invested to erect the same building with stabilized earth walls.

## 6. Conclusions

Analyzing the results obtained in the different phases of the lca, both for energy demand and emissions, it can be stated that the manufacturing phase of the construction materials is the most relevant chapter for both for cumulative energy demand and global warming potential of a building’s embodied energy. Since this is the most relevant phase, determinations or modifications of it are fundamental, and so it is the material choice during the design phase of a building. According to the results obtained, approximately three SW homes could be erected with the same amount of CO_2_ emissions and cumulative energy demand for every two homes built with BW.

Future possible research directions that would improve or expand this work are related to the comparison with other types of construction solutions, not only for load-bearing walls, but for envelope blocks without structural function. Other building types, other climates, and geographical conditions could be evaluated. The potential recyclability of these materials could also be assessed, something that is not reflected in the limits of this LCA.

## Figures and Tables

**Table 1:** Thickness of the load-bearing walls and insulation thickness for the different levels.

External Wall Type	Building Level	Wall Thickness (m)	Insulation Thickness (m) PUR	
			LOCATION I	LOCATION II
Ceramic Brick (BW)	Level 3	0.250	0.02	0.04
	Level 2	0.250	0.02	0.04
	Level 1	0.375	0.01	0.03
Concrete Block (CW)	Level 3	0.200	0.02	0.04
	Level 2	0.200	0.02	0.04
	Level 1	0.400	0.02	0.04
Stabilized Soil (SW)	Level 3	0.300	0.02	0.04
	Level 2	0.350	0.02	0.03
	Level 1	0.450	0.01	0.03

**Table 2:** Energy use and greenhouse gas emissions (GWP) for 1 tone merino wool top as reported by Barber and Pellow [40].

Impact Category	On Farm	Processing	Transport	Total
Total energy (MJ/t wool top)	22,550	21,700	1490	45,730
GWP (T CO_2_-eq/t wool top)	1655	471	103	2229

**Table 3:** Inventory list of the materials used and corresponding name in the ECOINVENT database. Unitary values for Global Warming Potential and Embodied Energy.

Component	ID	Name (ECOINVENT)	Unit	Global Warming Potential 100a CML 2001 kg CO_2_-eq	Embodied Energy Cumulative Energy Demand MJ
Gravel	464	gravel, round, at mine	kg	0,0024	0,0576
Limestone	466	limestone, at mine	kg	0,0019	0,0283
Concrete	504	concrete, normal, at plant	m^3^	265,2200	1447,2335
Bricks	512	ceramic tiles, at regional storage	kg	0,7651	14,9540
Plaster Board	517	gypsum plaster board, at plant	kg	0,3600	6,2652
Mortar	537	cement mortar, at plant	kg	0,1953	1,5182
Clay plaster	538	clay plaster, at plant	kg	0,0195	0,5181
Glass	805	flat glass, coated, at plant	kg	0,6855	15,0414
Aluminium	1059	aluminum, secondary, from new scrap, at plant	kg	0,4102	8,4313
Reinforcing steel	1141	reinforcing steel, at plant	kg	1,3442	20,9352
Steel	1154	steel, low-alloyed, at plant	kg	1,6294	26,1590
Paint	1670	alkyd paint, white, 60% in solvent, at plant	kg	2,5115	84,3073
Bitumen	1814	bitumen sealing, at plant	kg	1,0177	51,0742
Polyethylene	1829	polyethylene, HDPE, granulate, at plant	kg	1,8921	79,8534
Polyurethane	1838	polyurethane, flexible foam, at plant	kg	4,4208	101,2692
Polyvinylchloride	1840	polyvinylchloride, at regional storage	kg	2,1625	59,0158
Rubber	1847	synthetic rubber, at plant	kg	3,1972	101,3493
Water	2288	tap water, at user	kg	0,0003	0,0062
Wood	2507	sawn timber, softwood, planed, kiln dried, at plant	m^3^	713,1300	12792,1890
Sand	464	gravel, round, at mine (Tierra)	kg	0,0024	0,0576
Algae *	A001	Algae, at regional storehouse	kg	0,0200	20,0000
Sheep Wool **	P001	Wool mat, at plant	kg	0,9850	13,4200
Electricity (Const-Dem)	698	electricity mix	kWh	0,5004	10,9038
Diesel (Const-Dem)	559	diesel, burned in building machine	MJ	0,0910	1,3799
Transport (Const-Dem)	1943	transport, lorry 32 t	tkm	0,1663	2,8149
Electricity (Operational)	698	electricity mix	kWh	0,5004	10,9038
Natural Gas (Operational)	1327	natural gas, high pressure, at consumer	MJ	0,0101	1,1359
Brick final disposal	2005	disposal, building, brick, to final disposal	kg	0,0141	0,3110
Concrete final disposal	2007	disposal, building, cement (in concrete) and mortar, to final disposal	kg	0,0148	0,3217
Glass final disposal	2019	disposal, building, glass sheet, to final disposal	kg	0,0108	0,2614
Polyurethane final disposal	2040	disposal, building, polyurethane foam, to final disposal	kg	2,4699	1,3770
Polyvinylchloride final disposal	2043	disposal, building, polyvinylchloride products, to final disposal	kg	2,2223	12,5207
Steel final disposal	2048	disposal, building, reinforcement steel, to final disposal	kg	0,0678	1,1252
Wood final disposal	2052	disposal, building, waste wood, untreated, to final disposal	kg	1,4743	0,2038
Aluminium final disposal	2090	disposal, aluminium, 0% water, to municipal incineration	kg	0,0369	0,7668
Bitumen final disposal	2217	disposal, bitumen, 1.4% water, to sanitary landfill	kg	0,1079	0,3407
Inert material final disposal	2221	disposal, inert material, 0% water, to sanitary landfill	kg	0,0128	0,3330

* Data provided by Resurreccion *et al.* [41]. ** Data provided by Barber and Pellow [40].

**Table 4:** Inventory list of the materials used for every type of building system and climate.

Stage	Component	Unit	BW		CW		SW	
			LOCATION I	LOCATION II	LOCATION I	LOCATION II	LOCATION I	LOCATION II
Manufacture PH_1_	Gravel	kg	199.316,16	199.316,16	199.316,16	199.316,16	199.316,16	199.316,16
	Limestone	kg	776,96	776,96	776,96	776,96	776,96	776,96
	Concrete	m^3^	158,36	158,36	185,97	185,97	158,36	158,36
	Bricks	kg	196.924,82	196.924,82	83.044,34	83.044,34	83.044,34	83.044,34
	Plaster Board	kg	10.071,90	10.071,90	10.071,90	10.071,90	10.071,90	10.071,90
	Mortar	kg	86.316,30	86.316,30	20.461,47	20.461,47	10.446,01	10.446,01
	Clay plaster	kg	19.666,60	19.666,60	19.666,60	19.666,60	19.666,60	19.666,60
	Glass	kg	3.583,13	3.583,13	3.583,13	3.583,13	3.583,13	3.583,13
	Aluminium	kg	2.329,44	2.329,44	2.329,44	2.329,44	2.329,44	2.329,44
	Reinforcing steel	kg	6.550,74	6.550,74	6.550,74	6.550,74	6.550,74	6.550,74
	Steel	kg	314,62	314,62	314,62	314,62	314,62	314,62
	Paint	kg	8.263,95	8.263,95	8.263,95	8.263,95	8.263,95	8.263,95
	Bitumen	kg	258,39	258,39	258,39	258,39	258,39	258,39
	Polyethylene	kg	45,81	45,81	45,81	45,81	45,81	45,81
	Polyurethane	kg	155,93	27,46	187,11	467,78	155,93	311,85
	Polyvinylchloride	kg	27,46	27,46	27,46	27,46	27,46	27,46
	Rubber	kg	79,06	79,06	79,06	79,06	79,06	79,06
	Water	kg	34.664,05	34.664,05	26.845,78	26.845,78	101.112,30	101.112,30
	Wood	m^3^	4,11	4,11	4,11	4,11	4,11	4,11
	Sand	kg	0,00	0,00	0,00	0,00	226.324,80	226.324,80
	Algae	kg	0,00	0,00	0,00	0,00	2.715,90	2.715,90
	Sheep Wool	kg	0,00	0,00	0,00	0,00	565,81	565,81
Construction and Demolition PH_2_	Electricity (Const-Dem)	kWh	34.058,76	34.501,07	29.467,66	30.131,13	31.651,75	32.762,28
	Diesel (Const-Dem)	MJ	286.093,61	289.808,99	247.528,31	253.101,48	29.737,79	275.203,15
	Transport (Const-Dem)	tkm	111.348,48	111.370,00	97.721,92	97.754,20	94.982,97	97.186,61
Operational PH_5_	Electricity (Operational)	kWh	767.477,57	814.227,25	767.477,57	814.227,25	767.477,57	814.227,25
	Natural Gas (Operational)	MJ	3.867.080,73	7.658.781,91	3.867.080,73	7.658.781,91	3.867.080,73	7.658.781,91
Final Disposal PH_4_	Brick final disposal	kg	196.924,82	196.924,82	83.044,34	83.044,34	83.044,34	83.044,34
	Concrete final disposal	kg	395.893,90	395.893,90	464.924,50	464.924,50	395.893,90	395.893,90
	Glass final disposal	kg	3.583,13	3.583,13	3.583,13	3.583,13	3.583,13	3.583,13
	Polyurethane final disposal	kg	155,93	343,04	187,11	467,78	155,93	311,85
	Polyvinylchloride final disposal	kg	27,46	27,46	27,46	27,46	27,46	27,46
	Steel final disposal	kg	6.865,36	6.865,36	6.865,36	6.865,36	6.865,36	6.865,36
	Wood final disposal	kg	2.874,80	2.874,80	2.874,80	2.874,80	2.874,80	2.874,80
	Aluminum final disposal	kg	2.329,44	2.329,44	2.329,44	2.329,44	2.329,44	2.329,44
	Bitumen final disposal	kg	258,39	258,39	258,39	258,39	258,39	258,39
	Inert material final disposal	kg	324.671,08	324.671,08	258.816,25	258.816,25	478.407,30	478.407,30

**Table 5:** Mean environmental impact values showed by type of building construction system and climate location.

Total Results		Global Warming Potential (kg CO_2_-eq /m^2^ year				Cumulative Energy Demand (MJ-eq/m^2^ year)			
		PH1, PH4	PH2, PH3	Total Embodied	Total Operational PH5	PH1, PH4	PH2, PH3	Total Embodied	Total Operational PH 5
LOCATION I	Ceramic Bricks Wall (BW)	264.949,39	61.586,88	326.536,27	422.971,93	11.833,84	2.447,38	14.281,22	12.760.933,10
	Concrete Blocks Wall (CW)	171.385,34	53.515,04	224.900,38	422.971,93	8.014,74	2.104,76	10.119,50	12.760.933,10
	Stabilized Soil Wall (SW)	164.542,52	34.340,63	198.883,15	422.971,93	8.182,94	2.214,66	10.397,61	12.760.933,10
LOCATION II	Ceramic Bricks Wall (BW)	266.238,71	63.256,98	329.495,69	484.546,96	16.693,07	2.411,88	19.104,95	17.577.594,30
	Concrete Blocks Wall (CW)	174.206,50	54.115,19	228.321,69	484.546,96	8.106,53	2.139,21	10.245,74	17.577.594,30
	Stabilized Soil Wall (SW)	168.551,38	56.449,75	225.001,13	484.546,96	8.219,03	2.233,47	10.452,50	17.577.594,30
**Results per m^2^ and year**		**PH1, PH4**	**PH2, PH3**	**Total Embodied**	**Total Operational PH5**	**PH1, PH4**	**PH2, PH3**	**Total Embodied**	**Total Operational PH 5**
LOCATION I	Ceramic Bricks Wall (BW)	11,95	2,78	14,73	19,07	0,53	0,11	0,64	575,48
	Concrete Blocks Wall (CW)	7,73	2,41	10,14	19,07	0,36	0,09	0,46	575,48
	Stabilized Soil Wall (SW)	7,42	1,55	8,97	19,07	0,37	0,10	0,47	575,48
LOCATION II	Ceramic Bricks Wall (BW)	12,01	2,85	14,86	21,85	0,75	0,11	0,86	792,69
	Concrete Blocks Wall (CW)	7,86	2,44	10,30	21,85	0,37	0,10	0,46	792,69
	Stabilized Soil Wall (SW)	7,60	2,55	10,15	21,85	0,37	0,10	0,47	792,69

## Abbreviations

The following abbreviations are used in this manuscript:

LCA	Life Cycle Assessment
OE	Operational energy
EE	Embodied Energy
BW	Fired clay brick masonry walls
CW	Concrete block masonry walls
SW	Stabilized soil block masonry walls (SW)
GWP	Global Warming Potential
PH1	Manufacturing phase
PH2	Construction & Demolition phase
PH3	Transport phase
PH4	Final Disposal
PH5	Operational energy. 5 years lifespan
